# Performance of Miniature Carbon Nanotube Field Emission Pressure Sensor for X-Ray Source Applications

**DOI:** 10.3390/mi16070817

**Published:** 2025-07-17

**Authors:** Huizi Zhou, Wenguang Peng, Weijun Huang, Nini Ye, Changkun Dong

**Affiliations:** 1 Wenzhou Key Lab of Micro-Nano Optoelectronic Devices, Wenzhou University, Wenzhou 325035, China; 22451026021@stu.wzu.edu.cn (H.Z.); hwj@wzu.edu.cn (W.H.); 22451026017@stu.wzu.edu.cn (N.Y.); 2 Kailong Medical Instruments Co., Ltd., Hangzhou 311401, China; pengwenguang@radiiray.com

**Keywords:** multi-walled carbon nanotube, field emission, adsorption, pressure sensor, X-ray tube

## Abstract

There is a lack of an effective approach to measure vacuum conditions inside sealed vacuum electronic devices (VEDs) and other small-space vacuum instruments. In this study, the application performance of an innovative low-pressure gas sensor based on the emission enhancements of multi-walled carbon nanotube (MWCNT) field emitters was investigated, and the in situ vacuum performance of X-ray tubes was studied for the advantages of miniature dimension and having low power consumption, extremely low outgassing, and low thermal disturbance compared to conventional ionization gauges. The MWCNT emitters with high crystallinity presented good pressure sensing performance for nitrogen, hydrogen, and an air mixture in the range of 10^−7^ to 10^−3^ Pa. The miniature MWCNT sensor is able to work and remain stable with high-temperature baking, important for VED applications. The sensor monitored the in situ pressures of the sealed X-ray tubes successfully with high-power operations and a long-term storage of over two years. The investigation showed that the vacuum of the sealed X-ray tube is typical at a low 10^−4^ Pa level, and pre-sealing degassing treatments are able to make the X-ray tube work under high vacuum levels with less outgassing and keep a stable high vacuum for a long period of time.

## 1. Introduction

Vacuum electronic devices (VEDs), including microwave tubes, X-ray tubes, and vacuum power amplifiers, operate under high voltage and current conditions and are key components in fields of telecommunications, healthcare, broadcasting, and industrial detections [1,2,3,4,5]. The vacuum performance directly impacts the operational characteristics of these devices, including the electron emission stability and cathode lifetime. Operating at lower vacuum levels not only destabilizes electron emissions and shortens device lifetime but also brings about particle interference with the light and electron beams, significantly degrading device performance [6,7]. A high-vacuum environment will minimize particle collisions and reduce the erosion of electron emitters from gas adsorptions, thereby extending the device lifetime [8]. Therefore, the vacuum condition is a key factor to reach high operation performance for VEDs. Consequently, the real-time measurement of internal vacuum conditions is crucial not only to enable a lifetime assessment but also to facilitate the timely identification of technical states for the sealed devices. There are various types of vacuum gauges operating in wide pressure ranges [9,10,11,12,13], for example, Han et al. [14]’s proposed miniature capacitance diaphragm gauge capable of systematically measuring rough- and medium-vacuum areas with a range of 0.1 Pa to 84 kPa (atmospheric pressure); Wang et al. [15]’s MEMS-based friction vacuum gauge achieving measurements from 1.6 × 10^−3^ Pa to 0.1 Pa; and Lin et al. [16]’s MEMS Pirani vacuum gauge enabling wide-range detection spanning 4 Pa to 10^5^ Pa. However, there is still a lack of miniaturized high-vacuum-compatible sensors that can be integrated into narrow vacuum devices. Furthermore, driven by technological advancements and new application demands, vacuum equipment and measurement instruments are trending toward miniaturization and portability, highlighting the significance of developing reliable miniature pressure sensors [17].

Carbon nanotubes (CNTs) possess an excellent specific surface area property [18,19,20], benefiting interactions with gas molecules to improve sensor sensitivity [21]. Their superior electrochemical properties and high electron mobility facilitate rapid responses to environmental changes [22], making real-time detections of gas or chemical concentration variations possible. Additionally, CNTs exhibit stable chemical properties [23,24] and corrosion resistance [25,26], ensuring reliable performance in harsh environments, critical for long-term applications. The microscale feature of CNTs favors the development of miniature sensors to meet the growing demands for compact and portable electronic devices [27,28,29,30]. Different types of miniature pressure sensors have been developed, as shown in Table 1. In contrast, our sensor enables the pressure detection in higher vacuum regime. However, this sensor is specifically designed for compact high-vacuum devices and operates effectively within ultra-high-vacuum (UHV) and high-vacuum (HV) regimes. It struggles to measure low-vacuum and rough-vacuum ranges, demonstrating limitations in achieving broader measurement coverage.

Our group has developed a low-pressure gas sensing technique based on CNT field emission enhancement with the advantages of small dimension, wide measurement range, low power consumption, and cost-effectiveness [35,36,37]. Building upon prior development, this study focuses on the controllable fabrication of sensor cathodes and investigation of application performance for VEDs, including the field emission performance, sensing properties for different gases, sensing reliability, and X-ray tube applications. This research is expected to promote the application of the novel CNT-based miniature low-pressure sensor for in situ vacuum measurement in X-ray tubes and other VEDs.

## 2. Materials and Methods

The multi-walled carbon nanotube (MWCNT) cathode was synthesized by thermal chemical vapor deposition (CVD) directly on the Ni alloy substrate [38]. At first, the substrate underwent an anodization treatment (oxalic acid solution: 18.9 g/500 mL, 150 V DC, 1 min anodization) to enhance its specific surface area, facilitating the characteristic performance of MWCNT cathodes with superior field emission performance and high sensing capability. The anodization treatment generates uniformly distributed nanoscale protrusions and recesses on the cathodic metal surface. This nanostructured substrate enables the preparation of carbon nanotube materials with exceptional adhesion properties. Crucially, this approach achieves two key advantages without altering the fundamental characteristics of the metal surface, namely (1) a significant enhancement of the specific surface area and (2) the creation of a metal substrate with a highly uniform surface morphology. The subsequent growth of carbon nanotubes on such anodized substrates yields materials with improved macroscopic uniformity, demonstrating both consistent spatial distribution and remarkably uniform tube diameters across the sample. The anodized substrate was sequentially cleaned with acetone, anhydrous ethanol, and deionized water, followed by MWCNT synthesis in a three-zone CVD furnace. During the growth, argon served as both the carrier and protective gas, while acetylene was utilized as the carbon source gas. The MWCNT film was synthesized at 750 °C under 8 Torr for 25 min. The as-prepared MWCNT cathode material was systematically characterized using transmission electron microscopy (TEM, Titan G2 60–300, FEI, Hillsboro, OR, USA), scanning electron microscopy (SEM, JSM-7100F, JEOL, Tokyo, Japan), and Raman spectroscopy (DXR3, Thermo Scientific, Waltham, MA, USA) to analyze its morphological and crystallographic properties. Previous studies showed that CNTs with excellent hydrogen and nitrogen sensing characteristics typically demonstrate high structural crystallinity. Raman spectral analysis was conducted to further investigate the relation between sensing performance and crystallinity under various application conditions.

The MWCNT film was employed as the field emission cathode of the sensor, and its field emission and sensing performance were tested in a high-vacuum turbo system. Prior to testing, the system was baked at 250 °C for 12 h to achieve a high-vacuum background at the 10^−7^ Pa level. The sensor was designed as a diode configuration, with an MWCNT film of 5 mm^2^ in area as the cathode and a molybdenum plate as the anode at an inter-electrode spacing of 300 μm. By adjusting an ultra-high-vacuum needle valve, the system pressure was precisely controlled to perform field emission current measurements under varying pressures, thereby evaluating the sensing performance. The sensing mechanism relies on the gas adsorption-induced enhancement of the field emission current [35]. Taking hydrogen as an example, hydrogen atoms from field emission Joule heating-assisted dissociation chemisorb on CNT surfaces. Due to the stronger electronegativity of carbon, surface dipoles with positive-charged H would be formed. These dipoles facilitate the electron extraction from the CNT, resulting in the reduction in effective work function. As a result, the field emission current will be enhanced. Theoretical and field emission electron energy distribution (FEED) investigations revealed that the effective work functions of the CNT emitter decrease with the increase of H_2_ adsorption coverage, which is related to the partial pressures of the sensing species [39]. Therefore, the emission current increase rates are getting bigger with the pressure increase. The sensor is normally operated under low-emission currents at the micron ampere level to maximize the enhancement effect, because high-emission currents could desorb gas adsorbents by Joule heating. To improve the measurement instability due to low current variations, a multi-point current acquisition method was implemented for a certain time interval, typically 2–5 min, and then collected data was summed and averaged to obtain stable and reproducible sensing currents [35].

During the test, the field emission characteristic performance of the sensor cathode was first evaluated to optimize the parameters of the operation voltages. Before the sensing current measurement, the CNT cathode was applied to a field emission Joule-heating degassing process for 2 min to achieve a clean emitter surface. Subsequently, the cathode emission current was stabilized at an initial sensing current of 1 μA, followed by a 5 min emission period under constant voltage. Then multiple current values under a fixed interval, normally 0.5 s, were recorded during this period. Finally, the sensing current (*I_N_*) at a specific pressure was achieved by averaging all current data: $I_{N}=\Sigma_{i=1}^{120N}\frac{I_{i}}{120N}$, where *N* represents the test duration of each pressure in a minute. According to this process, a sensor controller was developed to perform the actual measurement. Then the sensing calibration curve was obtained with the sensing currents against the corresponding pressures. In the application performance investigation, the sensor was integrated within the X-ray tube to monitor the internal vacuum during sealing-off and under various operation conditions.

## 3. Results and Discussion

**Figure 1:**
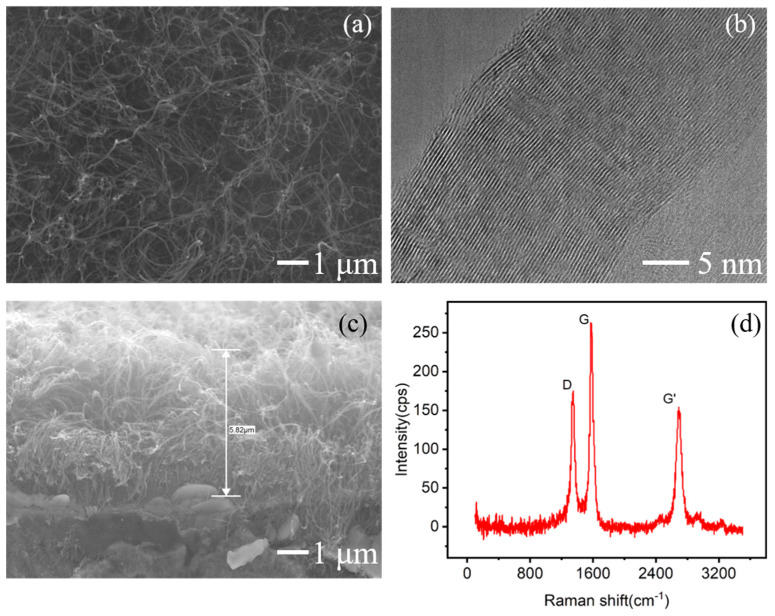
MWCNT emitters grown on alloy substrate. (**a**) SEM top view, (**b**) TEM image, (**c**) SEM side view, and (**d**) Raman spectra with I_D_/I_G_ = 0.665.

As shown in Figure 1, SEM and TEM images reveal that the MWCNTs were uniformly distributed on the substrate surface, with diameters ranging from 50~60 nm and approximately 60~70 concentric tubular layers. A cross-sectional view indicates that the MWCNT film had a thickness of 5~6 μm. The Raman spectroscopy analysis demonstrated that the intensity ratio of the disorder-induced peak (I_D_) to the graphite peak (I_G_) is 0.665, suggesting high crystallinity of the MWCNT emitter with less disordered or amorphous carbon impurities.

**Figure 2:**
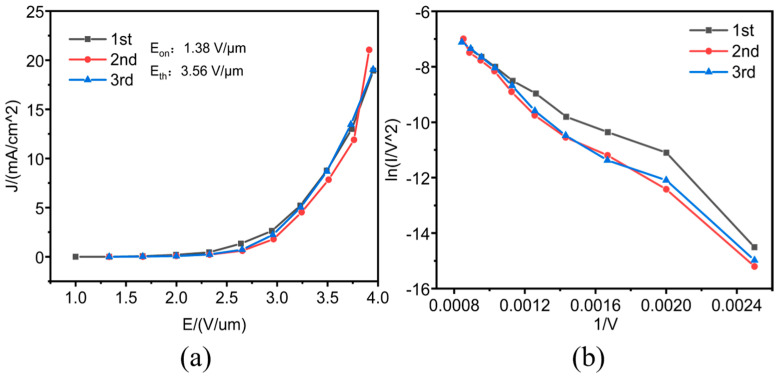
Field emission performance of MWCNT cathode. (**a**) J-E curve, (**b**) F-N curve.

The field emission characteristic curves of the MWCNT cathode are presented in Figure 2, exhibiting a turn-on field (at a current density of 10 μA/cm^2^) of 1.38 V/μm and a threshold field (at 10 mA/cm^2^) of 3.56 V/μm. The relatively low values of both turn-on and threshold fields demonstrate the excellent field emission performance of emitters, enabling low-voltage operations and benefiting the long lifetime of the sensor. The cathode presented excellent emission reproducibility with the second and third J-E curves overlapping greatly. The Fowler–Nordheim (F-N) plots reveal a distinct deviation of the first test curve from the following two curves. This phenomenon can be attributed mainly to two factors. Firstly, gas molecules adsorbed on the CNT surface enhanced field electron emissions during the initial test cycle [40,41], and this effect faded away in the following test cycles after the gas desorption from high-emission Joule heating. Secondly, longer CNTs preferentially emitted electrons from length-dependent emission priority under lower electric fields with typical local “hot” emission spots, which also died out after high current emissions, leading to the homogenization of emission sites. Consequently, the emission approached to intrinsic F-N characteristics in subsequent tests, demonstrating the self-stabilizing nature of the CNT cathodes under repeated operational conditions.

**Figure 3:**
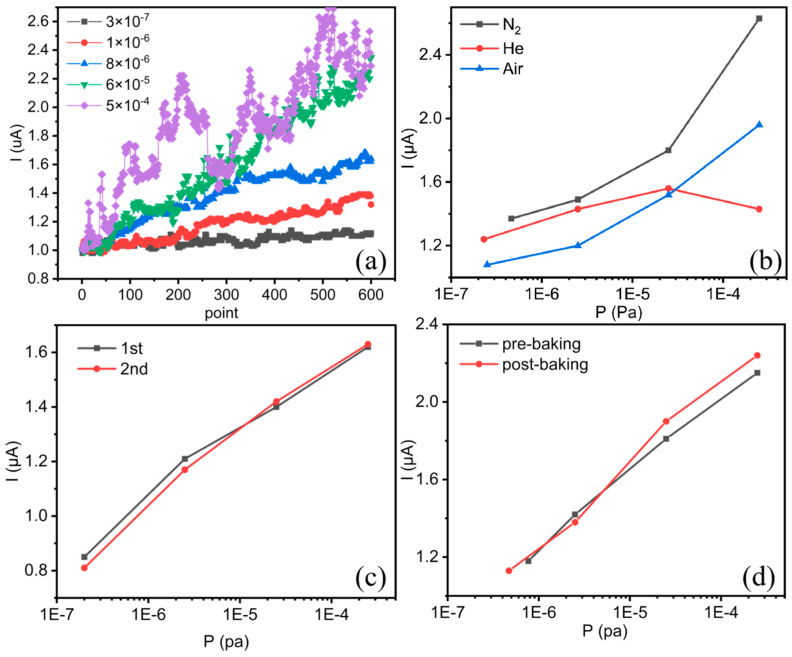
MWCNT sensing performance tests: (**a**) emission current climbs under different nitrogen pressures; (**b**) sensing response under different atmospheres; (**c**) nitrogen sensing performance repeatability test in vacuum system; (**d**) comparison of sensing curves before and after baking.

The pressure sensing is based on the emission enhancement effect in a low current range [35]. As shown in Figure 3a, under an initial current of 1 μA, when the system pressure was adjusted from 3 × 10^−7^ Pa to 5 × 10^−4^ Pa, the current increase rates jumped from 11% to 145% in 5 min, demonstrating a pronounced pressure-dependent sensing response.

The gas sensing behaviors were investigated under various low-pressure gas environments, including nitrogen, helium, and an air mixture. As illustrated in Figure 3b, the sensor based on MWCNTs of I_D_/I_G_ < 0.8 demonstrated significant gas sensing responses in the pressure range of 1 × 10^−7^ to 2.5 × 10^−4^ Pa. The sensing currents increase stably with pressure for nitrogen gas and air, a crucial behavior for low-pressure sensing applications. In contrast, there is no stable sensing response for helium. Previous investigations revealed that stable sensing responses could be achieved for hydrogen and nitrogen for CNT cathodes with high crystallinities (typically I_D_/I_G_ < 0.8) [37,42], whereas CNT emitters with low crystallinities (I_D_/I_G_ > 0.9) may show significant helium responses [36]. According to the first-principles investigations, such a sensing performance difference originates from different gas–CNT reaction mechanisms. Nitrogen generally adsorbs physically on the CNT surface, generating a potential well at the adsorption site that reduces the electron tunneling energy barrier, thus facilitating electron emission. In contrast, helium adsorption occurs preferentially at CNT defect sites, resulting in charge accumulation to enhance the emission current. Figure 3c presents two nitrogen pressure sensing curves from two consecutive test cycles, indicating that the sensor possessed excellent measurement reproducibility. As VEDs typically undergo high-temperature outgassing treatment prior to sealing-off to minimize the outgassing effect during high-energy operations, we conducted a high-temperature baking experiment under 420 °C for 16 h on the sensor in a vacuum furnace to evaluate the consistencies of the field emission, structural, and sensing performances, as shown in Figure 3d. The sensing currents after baking remained consistent with deviations of less than 5%, demonstrating good sensing stability.

**Figure 4:**
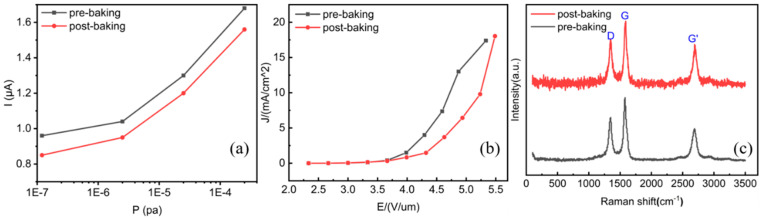
Relations of sensing performance decline with field emission and structural properties after high-temperature baking: (**a**) sensing curves before and after baking; (**b**) J-E curve before and after baking: pre-baking: E_on_ = 2.69 V/μm, E_th_ = 4.72 V/μm; post-baking: E_on_ = 2.7 V/μm, E_th_ = 5.24 V/μm; (**c**) Raman spectra before and after baking: pre-baking: I_D_/I_G_ = 0.679; post-baking: I_D_/I_G_ = 0.708.

Even though the sensor exhibited reliable sensing performance with high-temperature treatments, there exists larger attenuations on sensing performance for some sensors. As shown in Figure 4, the sensing currents decreased by about 7% post-baking for a sample, while the field emission performance turn-on and threshold fields increased from 2.69 V/µm to 2.7 V/µm and from 4.72 V/µm to 5.24 V/µm, respectively, indicating a slight decline in field emission performance. Raman analysis showed that the I_D_/I_G_ ratio increased from 0.679 to 0.708, implying the deterioration of the CNT crystallinity in this process. The high-temperature baking could introduce additional defect sites, degrading the crystallinity of CNTs [43]. Furthermore, the contact resistance [44] and electrical resistance [45] could increase. All these factors will lead to the deterioration of field emissions and, consequently, a decline in sensing performance.

**Figure 5:**
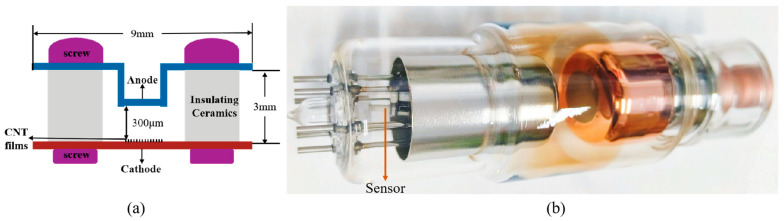
Application performance test: (**a**) sensor structure diagram; (**b**) X-ray tube sealed with sensor.

**Figure 6:**
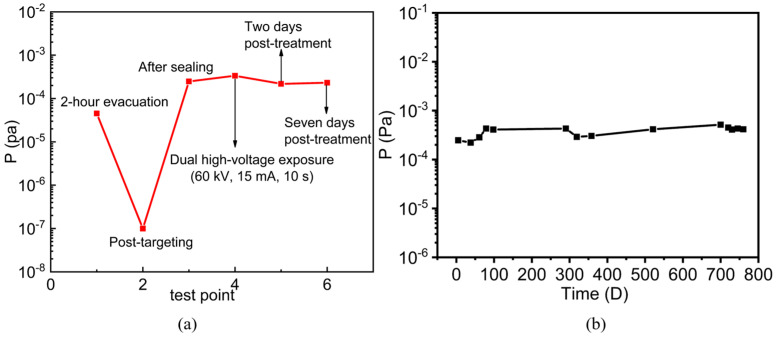
In situ vacuum of X-ray tubes by CNT miniature sensor: (**a**) before and after sealing-off; (**b**) long-term storage period.

To test the in situ vacuum performance, practical sensors were fabricated using selected MWCNT cathodes with good sensing effects, as illustrated in Figure 5a. Generally, the main residual gases inside X-ray tubes include H_2_ and N_2_. Based on the good sensing characteristics of the MWCNT sensor to these gases and the air mixture, the sensor is anticipated to monitor the pressures inside the X-ray tubes effectively. The sensor was integrated beneath the thermionic cathode, as illustrated in Figure 5b. The in situ vacuum measurements were conducted at several critical operational phases, including post-static evacuation, post-target bombardment before sealing-off, after sealing-off, post-operation (60 kV and 15 mA for 10 s), and during the static storage state. The corresponding measurement results are presented in Figure 6a.

The vacuum of the X-ray tube reached 4.52 × 10^−5^ Pa after 2 h of static evacuation. However, the vacuum level was improved significantly to the lower 10^−7^ Pa level following the cathode and anode bombardments, with electrode temperatures of around 600 °C during the bombardment. This demonstrates the critical role of high-temperature bombardment in enhancing vacuum performance. Then the tube was sealed, and the CNT sensor measured a vacuum of 2.48 × 10^−4^ Pa, showing the significant drop in vacuum. After the dual high-voltage operation (60 kV, 15 mA tube current, 10 s), the pressure increased to 3.35 × 10^−4^ Pa, slightly higher than the pressure in static mode. In addition, the pressures 3 days and 7 days after the sealing were 2.18 × 10^−4^ Pa and 2.31 × 10^−4^ Pa, respectively, indicating the recovery and stability of the vacuum after the operation. These measurements showed that the multiple pre-sealing degassing treatments are able to maintain the X-ray tube under a high vacuum level with less outgassing and pressure recovery capabilities after the operations.

To evaluate the long-term stability of the X-ray tube and the CNT sensor, the sensor was mounted inside another X-ray tube for vacuum measurements over a period of nearly 800 days, as shown in Figure 6b. Within the initial 60 days, the vacuum of the X-ray tube was in the low 10^−4^ Pa level ranging between 2 × 10^−4^ to 3 × 10^−4^ Pa. After 700 days, the pressure increased slightly to around the 4 × 10^−4^ Pa level, showing long-term vacuum stability. To confirm the reliability of the sensor, the vacuum measurements were conducted twice each day many times during this period, and the measurement deviations between two tests were less than 10%, demonstrating good measurement reproducibility. This investigation revealed the stable vacuum inside the glass X-ray tubes over a long period, and the long-term performance stability and reliability make the CNT pressure sensor a promising tool to measure in situ vacuum for sealed VEDs.

## 4. Conclusions

The application performance of a miniature MWCNT field emission pressure sensor was investigated in this study. The MWCNT emitters were grown on a nickel alloy substrate by chemical vapor deposition with excellent field emission properties. The MWCNT emitters with high crystallinity and typical Raman (I_D_/I_G_) ratios of less than 0.8 exhibited pressure sensing performance for nitrogen and an air mixture in the high-vacuum range. The miniature sensor demonstrated measurement reproducibility and stability under high-temperature impacts. The advantages of being miniature and having low power consumption, extreme low outgassing, and low thermal disturbance make the sensor applicable for in situ pressure measurements inside VEDs. The investigation revealed the vacuum performance inside X-ray tubes before and after sealing, which is important for the manufacturing and operation of X-ray products.

## Figures and Tables

**Table 1:** Critical parameters of carbon nanotube variants and comparative sensors.

Parameter	This Work	CNT Arrays	MWNT	SWCNT	MEMS Sensor (Yttrium Oxide)
Threshold field (V/μm)	3.56	2.3	/	/	/
Sensitivity	10^−7^–10^−3^ Pa	/	101–550 kPa	3%NO_2_–79.81%	1 × 10^−4^–100 Pa
Dimension parameter	4 × 9 mm^2^	1 cm^2^	6 × 6 mm^2^	2 × 2 cm^2^	12 × 12 × 3.3 mm^3^
References		[31]	[32]	[33]	[34]

## Data Availability

The original contributions presented in the study are included in the article, further inquiries can be directed to the corresponding author.
